# Documenting care together with patients: the experiences of nurses and patients

**DOI:** 10.1186/s12912-023-01309-6

**Published:** 2023-04-27

**Authors:** Helle Schøllhammer, Tina Magaard Jørgensen, Hanne Irene Jensen

**Affiliations:** 1 Day Surgery Unit, Kolding Hospital, Sygehusvej 24, Kolding, DK - 6100 Denmark; 2 Department of Regional Health Research, University of Southern Denmark, J. B. Winsløwsvej 19.3, Odense C, 5000 Denmark

**Keywords:** Patient participation, Patient involvement, Documentation, Medical records, Intervention

## Abstract

**Background:**

One way in which patients can participate in care is by taking part in medical documentation. Producing documentation together with patients has been found to decrease the amount of incorrect information, help patients to be involved, and promote shared decision-making. The aims of this study were to develop and implement a practice of producing documentation together with patients and to examine staff and patient experiences of this practice.

**Methods:**

A quality improvement study was conducted from 2019 to 2021 at a Day Surgery Unit in a Danish University Hospital. Before implementing a practice of documenting together with patients, nurses’ perceptions of doing documentation together with patients were examined via a questionnaire survey. After an implementation period, a similar follow-up survey with staff was conducted, together with structured patient phone interviews.

**Results:**

A total of 24 nursing staff out of 28 (86%) filled in the questionnaire at baseline and 22 out of 26 (85%) at follow-up. A total of 61 out of 74 invited patients (82%) were interviewed. At baseline, the majority (71-96%) of participants agreed that documentation done together with the patient would contribute to increased patient safety, fewer errors, real-time documentation, patient involvement, visible patient perspective, correction of errors, more accessible information and less duplication of work. At follow-up, significant decreases in the staffs’ positive perceptions of the benefits of documenting together with patients were found for all areas except for “real-time documentation” and “less duplication of work”. Almost all patients found it okay that the nurses wrote up medical documentation during the interview, and more than 90% of patients found the staff responsive or very responsive and present during the reception interview.

**Conclusion:**

Before implementation of a practice of documenting together with patients, the majority of staff assessed such documentation as being beneficial, but a significant decrease in positive assessment was found at follow-up, with challenges such as feeling less connected with the patient and practical, IT-related issues being described. The patients found the staff to be present and responsive and felt that it was important to know what was being written in their medical record.

## Background

Patient-centred care is an area of focus in healthcare around the world [1]. This form of care requires patients to be active partners in care [2], described with different terms such as participation, involvement, and engagement [2–4]. More than a decade ago, Sahlsted et al. defined patient participation in nursing care as “*an established relationship between nurses and patient, a surrendering of some power or control by the nurse, shared information and knowledge, and active engagement together in intellectual and/or physical activities*“ [4]. Patient participation covers a wide range of areas, from shared decision-making in treatment decisions at end-of-life [5] to participation in general nursing care [6], and it needs to be based on the individual patient’s preferences [7].

One area of patient participation in care is documentation in medical records written up together with patients. Such collaboration with patients has been found to decrease the amount of incorrect information, help patients and relatives to be involved, and promote shared decision-making [8, 9]. However, nurses face challenges with such documentation, such as lack of presence due to needing to pay attention to a computer while talking with the patient, technical problems related to the electronic patient records, work-related challenges such as time pressure, and challenges related to the patients’ medical conditions [9, 10]. A systematic review has identified that various factors, such as room layout, patient and provider styles of interaction, and strategies and techniques employed by clinicians, can determine whether interaction and communication are positive or negative [11].

The benefits of patient participation are reflected in the laws and regulations in several countries. It is a legal requirement in many Western countries, such as Canada, Norway, the USA and the Netherlands, to support patient participation in medical documentation and to secure patient access to healthcare documentation [9]. In Denmark, it is mandatory for the healthcare system to work systematically with patients and relatives [12]. Nevertheless, in a Danish University Hospital, patient surveys showed that many of the patients and their relatives did not feel involved in their treatment during hospitalization, and ward rounds were not always planned in such a way that relatives could participate. Documentation of care usually takes place at nursing offices or team stations, which is one of the reasons why patients and relatives are not always informed about plans and appointments (survey results not published).

Based on extant literature [8–11], patient surveys and accounts of (unpublished) experiences from other wards, the aims of this study were to develop and implement a practice of documenting together with patients and to examine the experiences of staff and patients with such a practice.

## Methods

### Design

A quality improvement study with pre- and post-tests. The study was performed from 2019–2021.

### Setting

A Danish Day Surgery Unit at a medium-sized University Hospital (app. 300 beds). Between 25 and 35 patients are treated daily within the specialties orthopedic surgery, gynecology and organ surgery (in 2022 in total 4,766). The nursing staff are involved in the entire patient process: reception of the patient, assistance with surgery and treatment, observation during recovery and discharge of the patient.

### Electronic patient record

The hospital use a joint electronic patient record (EPJ) system in which both physicians and nurses document. The day surgery nurses are responsible for documenting surgery facts such as: is the patient fasting, have they taken their usual medication and level of pain. Additionally, day surgery nurses are responsible for documenting that the patient has been informed about the day surgery process, that the patient has understood the information, and the patient’s preferences and specific problems, which may influence the surgery process and the ability of the patient to cope at home after the surgery. The nursing care and documentation is based on the nursing process (assessment, diagnosis, planning, implementation, and evaluation). However, the documentation does not always include all parts of the process but is a mix of checklists and prose text depending on the individual patient’s needs and care.

### Initiation of the study

The nursing staff from the Day Surgery Unit was presented with the project “Documentation with the patient” at a staff meeting, where all (28 nurses) were present. In this meeting, knowledge from literature [8–11] and other departments’ experiences about advantages and disadvantages of documenting together with patients were presented. After a discussion of benefits and challenges, a joint decision was made to go ahead with the study and to implement a practice in the unit of documenting together with patients.

### Participants

All nursing staff employed at the Day Surgery Unit and patients of 18 years of age or older, able to understand Danish and undergoing day surgery in the unit.

First, we wanted to examine the attitudes of the staff before developing and implementing a documentation practice together with the patient in the Day Surgery Unit. This was conducted via a questionnaire survey, which functioned as our baseline data. The baseline study also contributed to staff knowledge of the factors needed to implement a practice of producing documentation together with patients.

After the development and implementation of a practice of documenting together with patients, we conducted a follow-up study in which both staff and patients were asked to participate. After completion of the data analyses, the results of the study were presented at a staff meeting in the day surgery department. In this meeting, the staff could provide comments and experiences in relation to the “Documentation with patients” practice.

### Baseline

#### Staff questionnaire

The questionnaire survey was distributed among staff. The aim was to examine nursing staff members’ expectations and perceived benefits in regard to producing documentation together with patients, and to understand what is necessary for such documentation to be produced. The questionnaire was developed based on literature [8–11] and accounts from other departments that had already worked with a practice of writing documentation together with patients. The other departments’ experiences were that documenting together with patients predominantly was beneficial and that the experienced barriers all were connected to staff (concerns about lack of time, connection with the patient and uncertainty regarding the staff’s new role). The questionnaire consisted of 10 statements about possible benefits of writing documentation with patients, and the participants were asked about their level of agreement with the statements based on a five point Likert scale (strongly agree, agree, partly agree, disagree, strongly disagree).

Furthermore, the questionnaire included three open-ended questions where the participants were asked to describe further benefits, challenges and important factors for taking into account in relation to implementation of such a documentation practice.

The questionnaire was pilot tested by three staff members, and all statements were assessed as understandable and relevant. The pilot test was not included in the analysis, but the three staff members participated again in the main survey. The questionnaire was distributed electronically via the SurveyXact survey system in June 2019. All nursing staff received an e-mail with an invitation to participate in the survey and an individual link to the questionnaire (presented in Table 1). If no response was received, a reminder was sent after two weeks.

**Table 1:** Staff attitudes towards documentation with patients

Questions	Baseline (n = 24)		Follow-up (n = 22)		p-value^1^
To what extent do you agree that such documentation means: More patient safety; certainty that the right thing is documented. n(%)	Agree	23 (95.8)	Agree	17 (77.3)	< 0.001
	Partly agree	1 (4.2)	Partly agree	3 (13.6)	
	Disagree	0	Disagree	2 (9.1)	
To what extent do you agree that such documentation means: Gives the patient the opportunity to confirm / correct the documentation. n(%)	Agree	21 (87.5)	Agree	16 (72.7)	0.01
	Partly agree	2 (8.3)	Partly agree	5 (22.7)	
	Disagree	1 (4.2)	Disagree	1 (4.5)	
To what extent do you agree that such documentation means: More accurate documentation and fewer errors. n(%)	Agree	22 (91.6)	Agree	15 (71.4)	< 0.001
	Partly agree	2 (8.3)	Partly agree	4 (19.0)	
	Disagree	0	Disagree	2 (9.5)	
To what extent do you agree that such documentation means: More information available for the conversation with the patient. n(%)	Agree	17 (73.9)	Agree	16 (72.8)	0.003
	Partly agree	6 (26.1)	Partly agree	4 (18.2)	
	Disagree	0	Disagree	2 (9.1)	
To what extent do you agree that such documentation means: Real-time documentation. n(%)	Agree	23 (95.8)	Agree	20 (91.0)	0.08
	Partly agree	1 (4.2)	Partly agree	1 (4.5)	
	Disagree	0	Disagree	1 (4.5)	
To what extent do you agree that such documentation means: Less duplication of work. n(%)	Agree	17 (70.8)	Agree	13 (59.1)	0.17
	Partly agree	5 (20.8)	Partly agree	7 (31.8)	
	Disagree	2 (8.3)	Disagree	2 (9.1)	
To what extent do you agree that such documentation means: A more visible patient perspective. n(%)	Agree	20 (83.3)	Agree	14 (63.7)	< 0.001
	Partly agree	4 (16.7)	Partly agree	6 (27.3)	
	Disagree	0	Disagree	2 (9.1)	
To what extent do you agree that such documentation means: More patient involvement at the interview. n(%)	Agree	21 (87.5)	Agree	10 (45.4)	< 0.001
	Partly agree	2 (8.3)	Partly agree	10 (45.5)	
	Disagree	1 (4.2)	Disagree	2 (9.1)	
To what extent do you agree that such documentation means: More closeness in the relationship with the patient. n(%)	Agree	17 (70.8)	Agree	9 (40.9)	< 0.001
	Partly agree	3 (12.5)	Partly agree	10 (45.5)	
	Disagree	4 (16.7)	Disagree	3 (13.6)	

1. Chi square test or Fischer’s exact test.

#### “Documentation with the patient” practice

The new “Documentation with the patient” practice was inspired by other departments where the staff documented together with the patient, literature [8–11] and was furthermore based on the prerequisites the staff had drawn attention to in the baseline survey which included update of IT-equipment. The practice was developed by the two first authors.

Prior to the study, new patients were received in a conversation room, where, in accordance with the unit’s documentation guidelines, the nursing staff provided information about the Day Surgery Trajectory and made notes on paper about the information provided and about specific patient issues. These notes were then entered into the patients’ electronic patient records via computers in the staff office. In the new practice, documentation in the electronic patient records should be carried out with the patient during the reception interview in the conversation room. Based on the baseline study results, priority was given to the provision of portable laptops in the conversation rooms so that the patient and nurse could still sit opposite each other. A box was added to the electronic patient record for registration of documentation made together with the patient with the option of an explanation if this was not the case.

### Follow-up

#### Patient interviews

At a randomly picked two-week period in March 2021, all patients who fulfilled the inclusion criteria were invited to participate in a structured telephone interview the day after the surgery. The interview questions were validated questions from the Danish National Patient Satisfaction Survey [13] with an added opportunity for the patients to provide free comments. If the patients consented to participate, their name and phone number were registered, and before discharge from the day Surgery Unit, the patients received a paper version of the questions, so they knew what they would be asked about. The interviewer who contacted the patients was a quality improvement nurse who had not been involved in the care of the patients. In connection with the follow-up study, a journal audit confirmed that staff had documented together with all patients who participated in the interview study.

#### Staff questionnaires

The baseline staff questionnaire survey was repeated 18 months after implementation of the new documentation practice. In the follow-up survey, the three open-ended questions about benefits, challenges and necessary factors were replaced with one option for writing free-text comments.

### Data analysis

Quantitative data are presented with descriptive statistics (percentages). The Likert scale responses are presented in three groups: agree (strongly agree and agree), partly agree, and disagree (disagree and strongly disagree). The Chi-square test and the Fischer’s exact test (depending on cell numbers) were used to compare results between baseline and follow-up surveys. Qualitative data (questionnaire comments and patient responses) were analysed inspired by content analyses [14]. The comments were read a number of times by the first and second author, and together they coded and identified the main content. Afterwards, the comments and the main content was discussed with the third author.

The manuscript confers to the STROBE statement (Strengthening the Reporting of Observational studies in Epidemiology) [15].

## Results

### Staff questionnaires

A total of 24 nursing staff out of 28 (86%) filled in the questionnaire at baseline and 22 out of 26 (85%) at follow-up.

At baseline, more than 90% of the staff agreed that documentation in collaboration with the patient would contribute to increased patient safety, fewer errors and real-time documentation. More than 80% of staff agreed that documentation in collaboration with the patient would contribute to patient involvement, a more visible patient perspective, and correction of errors, and more than 70% of staff agreed that such documentation would contribute to closeness in the staff-patient relationship, more accessible information and less duplication of work (Table 1).

At follow-up, significant decreases in the staffs’ positive perceptions of the benefits of documenting together with patients were found for all areas except for “real-time documentation” and “less duplication of work” (Table 1).

### Comments from the staff baseline survey

In the baseline measurement, the free-text comments expanded on benefits and challenges.

Benefits of documenting in collaboration with the patient:

1. Less double documentation / real-time documentation.
2. Increased patient safety / correct documentation.
3. Increased patient involvement / patient perspective.

Challenges of documenting with patients.

1. More time-consuming.
2. Negative impact on nurse–patient relationship.
3. New workflows / competencies.
4. Physical framework / IT equipment.

### Comments from staff follow-up survey

In the follow-up study, the free-text comments mainly touched on (1) Patient relationship where the staff felt that the focus on the patient was disrupted, and (2) Difficulties in keeping focused during conversations with patients, where the staff found maintaining their concentration to be a challenge.

### Patient interviews

A total of 61 out of 74 invited patients (82%) were interviewed.

Almost all patients found that it was okay that the nurses documented in their medical record during the interview. More than 90% of the patients found the staff responsive or very responsive and present during the reception interview. About 50% of patients found it important or very important to know what was written in the journal, and more than 20% of the patients stated that they were not informed about what the staff wrote in the journal during the reception interview (Table 2).

**Table 2:** Patient experiences with documentation together with patients

Questions	Responses (n = 61)	
Did you find that the nursing staff was responsive to your needs? n(%)	Agree	60 (98.4)
	Partly agree	1 (1.6)
	Disagree	0
Did the staff ask about your own experiences with your illness / condition? n(%)	Agree	45 (73.8)
	Partly agree	9 (14.8)
	Disagree	7 (11.5)
Was it okay for the staff to write in your journal during the interview? n(%)	Agree	60 (98.4)
	Partly agree	1 (1.6)
	Disagree	0
Did the staff seem present at the interview in which they wrote in your journal? n(%)	Agree	55 (91.7)
	Partly agree	5 (8.3)
	Disagree	0
During the interview, were you informed of what the staff wrote in your journal? n(%)	Agree	34 (55.7)
	Partly agree	12 (19.7)
	Disagree	15 (24.6)
Is it important to know what the staff member writes in your journal during the interview? n(%)	Agree	31 (50.9)
	Partly agree	15 (24.6)
	Disagree	15 (24.6)
Did the staff encourage you to ask questions during the interview? n(%)	Agree	57 (93.5)
	Partly agree	4 (6.6)
	Disagree	0
Did the staff consider your needs when planning your discharge? n(%)	Agree	55 (90.1)
	Partly agree	5 (8.2)
	Disagree	1 (1.6)

### Comments from the patient study

The patient comments regarding the new documentation practice were predominantly positive.

One said, “It is important that it says the right thing in the medical record. There is some medicine I cannot tolerate and I was in doubt if it was in order” and another said: “It is very satisfactory and fine that it is written in the journal immediately - that way, you know that what is necessary will be done”. However, for a few, it was not important: “It is not so important to know what is written in the journal - I can read it afterwards”.

### Staff meeting

The results of the study were presented and discussed at a staff meeting. The main issues were that it had been difficult at first to have to document while the patient was present due to feelings of being less present, lack of computer skills and technical problems with IT equipment that delayed reception of patients. The staff requested more concrete and detailed instructions on how a reception, including documentation together with the patient, could take place. One comment was that if the patient just sits next to while the staff documents, this makes no difference in documentation practice. When discussing the results from the interview study with the patients, there was wide agreement among the staff that documentation, together with the patient, made sense.

## Discussion

In this study, we wanted to develop and implement a practice of producing documentation together with patients and to examine the experiences of staff and patients with such a practice. At baseline, the majority of nursing staff expected that documentation together with the patient would be beneficial in almost all areas. However, after implementation of the new practice, significant decreases in positive assessments were found. In the patient study though, this reservation was not found. The patients found the staff to be present and responsive. Furthermore, for more than half of the patients, it was important to know what was being written in their medical records.

The challenges described in the staff survey, such a feeling of connection with the patient being compromised and technical challenges with the electronic patient record, are consistent with results from similar surveys [11]. In a review study, computer use in the clinical encounter with the patient also showed a negative impact on the nurse-patient relationship [11]. It was difficult for staff to divide their attention between patient and computer and they felt a lack of presence due to needing to pay attention to a computer. Moreover, less information was shared with the computer than with paper medical records and the ability of patients to ask questions was negatively affected [11]. In our study, staff were used to use an electronic patient record, but not together with the patient. The staff found that the connection with the patient was disrupted when they documented on a computer while the patient was present. The staff also experienced a loss of focus. A similar finding was highlighted in a Dutch qualitative study conducted in 2021 [9]. In this study, community nurses were interviewed about documentation together with patients. Over half of the nurses in the study found it a challenge to document on a computer in collaboration with patients, as it felt uncomfortable interrupting the conversation in order to document on the computer [9]. However, in our study, patients did not feel that the attention of the staff was compromised.

Despite the challenges faced by the staff, the current study showed a high level of patient satisfaction and that documentation together with the patient was meaningful for the patients. The patients saw inclusion in documentation as a matter of course and stated that knowing what was written in their medical records mattered to them. Information sharing helps promote patient involvement [6], which is one of the key goals pursued by the healthcare system [12]. Therefore, documentation together with the patient is an important step in that direction. Such documentation provides an opportunity for the patient to immediately confirm or correct the information shared in the medical record. Likewise, information sharing improves the patient experience by making patients feel engaged, informed and respected [6].

However, 25% of the patients stated that they were not informed about what was actually written in their medical records during the reception interview. This suggests that although “Documentation together with the patient” had been registered for all participating patients, for a number of receptions, this had been documentation in the same room as the patient instead of documentation carried out with the patient.

Even though the staff at baseline agreed on changing the documentation practice to one of documentation together with the patient and requested technical equipment to be put in place, the follow-up survey showed that the implementation process lacked more concrete and detailed instructions on how documentation in collaboration with patients could actually take place [8, 9]. This is probably the main reason for the decrease in the staff’s positive assessments of the benefits of such documentation at follow-up. This is in line with Rogers’ theory of innovation, where a change of practice diffuses if the majority of participants do not adapt to the change [16]. Rogers describes five attributes of innovation that affect adoption: (1) relative advantage, (2) compatibility, (3) complexity, (4) trialability, and (5) observability [16]. In the current study, the nurses beforehand assessed the change of practice as advantageous and compatible with their practice. The practice developers assessed the new practice as simple, but in reality, the staff found it both technically and personally complex. Therefore, more detailed descriptions of how to implement the documentation in collaboration with patients and inclusion of innovators who could have provided peer training would have made matters easier. Likewise, the implementation process lacked triability where the new practice could be tried out in part, and observability where the process of the implementation with benefit could have been continuously followed, discussed and adjusted.

Other reasons for the decrease in the staff’s positive assessments of the benefits of such documentation could be the general challenge of accepting changes in practice, and the COVID-19 related practice changes may have contributed to this.

Even though to a certain extent the new documentation practice had diffused during the implementation period, the patient feedback rekindled the staff’s belief in the benefits of documentation together with the patient. If our study’s evaluation of such documentation had been limited to the experience of the staff, the conclusion of this project might have been quite different. This shows how important inclusion of the patient perspective is [17].

The strengths of the study include the high response rates and participation of both staff and patients in the evaluation. The main limitations of the study are the insufficient development and implementation processes. Furthermore, limitations include it being a small single-centre study and different levels of participants’ experience with the new practice, as some nurses only occasionally work with the reception of patients.

## Conclusion

Before implementation of a practice of writing up documentation together with patients, the majority of staff assessed such a practice as being beneficial, but a significant decrease in positive assessment was found at follow-up, with feelings of being less connected with patients and practical, IT-related issues being reported. The patients found the staff to be present and responsive and felt that it was important to know what was being written in their medical record. The implementation process would have benefitted from peer training and the provision of practical instructions on how to document in collaboration with patients. Presenting the positive patient feedback rekindled the staff’s belief in the benefits of documenting together with patients.

## Data Availability

The dataset generated and analysed during the current study are not publicly available due to participants’ confidentiality and the researchers have no ethical permission to share them. The corresponding author can be contacted if someone wants to request the data from the study.
